# High seroprevalence and risk factors for *Toxoplasma gondii* infection in Iquitos: A city in the Peruvian Amazon

**DOI:** 10.1371/journal.pntd.0014476

**Published:** 2026-11-10

**Authors:** Viviana Pinedo-Cancino, Sandra Chávez-Sarmiento, Oliver A. Bocanegra, Kary Vela-Tello, Edith Málaga-Machaca, Natalie M. Bowman, Maritza Calderón

**Affiliations:** 1 Laboratorio de Investigación de Productos Naturales Antiparasitarios de la Amazonía, Centro de Investigación de Recursos Naturales de la Amazonía, Universidad Nacional de la Amazonía Peruana, Iquitos, Loreto, Perú; 2 Facultad de Medicina Humana, Universidad Nacional de la Amazonía Peruana, Iquitos, Loreto, Perú; 3 Department of Microbiology & Immunology, School of Medicine, University of Texas Medical Branch, Galveston, Texas, United States of America; 4 Laboratorio de Investigación en Enfermedades Infecciosas, Laboratorios de Investigación y Desarrollo, Universidad Peruana Cayetano Heredia, San Martín de Porras, Lima, Perú; 5 Division of Infectious Diseases, University of North Carolina at Chapel Hill, Chapel Hill, North Carolina, United States of America; 6 Institute of Global Health and Infectious Diseases, University of North Carolina at Chapel Hill, Chapel Hill, North Carolina, United States of America; NHS Blood and Transplant, UNITED KINGDOM OF GREAT BRITAIN AND NORTHERN IRELAND

## Abstract

**Background:**

Toxoplasmosis, caused by the intracellular parasite *Toxoplasma gondii*, is a globally distributed infection with particularly high prevalence in tropical regions. Despite its high endemicity, population-based epidemiological data for the urban Amazon are scarce. This study aimed to determine the seroprevalence of *T. gondii* and identify sociodemographic and behavioral factors associated with *T. gondii* infection in the urban population of Iquitos, the largest city in the Peruvian Amazon.

**Methodology/principal findings:**

A cross-sectional study was conducted between July 11 and August 10, 2024. Serum samples were collected from 382 adults across four urban districts of Iquitos and tested using an in-house ELISA (sensitivity 98%, specificity 100%) for *T. gondii*-specific IgG antibodies. All 358 IgG-seropositive participants were tested for IgM antibodies. Bivariate and multivariable Poisson regression analyses with robust variance were performed to identify factors associated with seropositivity. The overall IgG seroprevalence was 93.7% (358/382; 95% CI: 90.8–95.9%), and IgM seroprevalence was 1.7% (6/358; 95% CI: 0.62–3.6%) among IgG-positive individuals. In multivariable analysis, age above 60 years (adjusted prevalence ratio [aPR]: 1.10; 95% CI: 1.04–1.17; p = 0.002), and monthly family income in the 372–593 USD bracket (aPR: 1.07; 95% CI: 1.01–1.15; p = 0.035) were independently associated with IgG seropositivity. Additionally, peri-urban/rural residence (aPR: 1.04; 95% CI: 1.01–1.08; p = 0.025) and use of river or rainwater as a drinking water source (aPR: 1.08; 95% CI: 1.02–1.14; p = 0.010) were independently associated with seropositivity, whereas living in brick/cement housing was negatively associated with seropositivity compared with wooden housing (aPR: 0.95; 95% CI: 0.91–0.99; p = 0.037), suggesting waterborne transmission and structural poverty as key environmental drivers of infection in this setting.

**Conclusions/significance:**

This study documents near-universal exposure to *T. gondii* in the urban population of Iquitos, underscoring toxoplasmosis as a major public health concern in the Peruvian Amazon. Age, income level, and peri-urban/rural residence were independent predictors of seropositivity. These findings support the prioritization of surveillance and prevention strategies targeting older adults and lower-income households in peri-urban areas, alongside environmental sanitation improvements.

## Author summary

We studied how common *Toxoplasma gondii* infection is among adults living in Iquitos, the largest city in the Peruvian Amazon and the only major city in the world inaccessible by road. This parasite spreads through contaminated food, water, or soil, and can cause serious illness in congenitally infected babies and people with weakened immune systems. We tested serum samples from 382 adults across the four city districts and found that 93.7% had been infected at some point in their lives one of the highest rates ever reported in a general urban population worldwide. About 2% of those infected also tested positive for IgM antibodies, which can persist for a long time and do not confirm recent or active infection. Older adults, people living in peri-urban or rural areas, and those with intermediate household incomes were more likely to have been infected. We also found that people who drink river or rainwater rather than treated or bottled water had a higher risk of infection, suggesting that contaminated water is an important route of transmission in Iquitos. Additionally, living in a wooden house, which is common in flood-prone peri-urban areas with limited sanitation, was independently associated with higher infection rates. Together, these findings point to structural environmental conditions inadequate water infrastructure and precarious housing as the primary drivers of this near-universal infection burden, rather than individual behaviors alone. Our results highlight the urgent need for continuous access to treated water, improved sanitation in peri-urban areas, and routine screening of pregnant women as priority public health actions to reduce the burden of this neglected disease in the Peruvian Amazon.

## Introduction

Toxoplasmosis is a globally distributed parasitic disease caused by the obligate intracellular protozoan *Toxoplasma gondii*, which infects a broad range of warm-blooded vertebrates, including humans [1]. An estimated one-third of the world’s population has been exposed to the parasite, with particularly high seroprevalence in tropical and subtropical regions [2,3]. The parasite’s life cycle involves felids as definitive hosts, which excrete environmentally resistant oocysts in their feces; humans acquire infection primarily through ingestion of tissue cysts in raw or undercooked meat, through consumption of food or water contaminated with oocysts [4,5]. While toxoplasmosis is asymptomatic in most immunocompetent individuals, it can cause severe and life-threatening complications in immunocompromised patients and, when acquired during pregnancy, may lead to congenital infection with neurological and ocular sequelae in the newborn [6,7].

The Loreto Region is the largest administrative region in Peru, covering approximately 368,852 km^2^ and entirely encompassed within the Amazon rainforest [8]. Its capital, Iquitos (Fig 1) with approximately 422,055 inhabitants [9], is the largest urban center in the Peruvian Amazon and is accessible only by river or air, which constrains access to centralized public health services and infrastructure. The city exhibits unique environmental and socioeconomic conditions that likely facilitate *T. gondii* transmission, including the convergence of domestic and sylvatic transmission cycles, limited access to potable water, inadequate sanitation infrastructure, substandard housing in peri-urban areas, and the frequent consumption of raw or undercooked meat.

**Fig 1:**
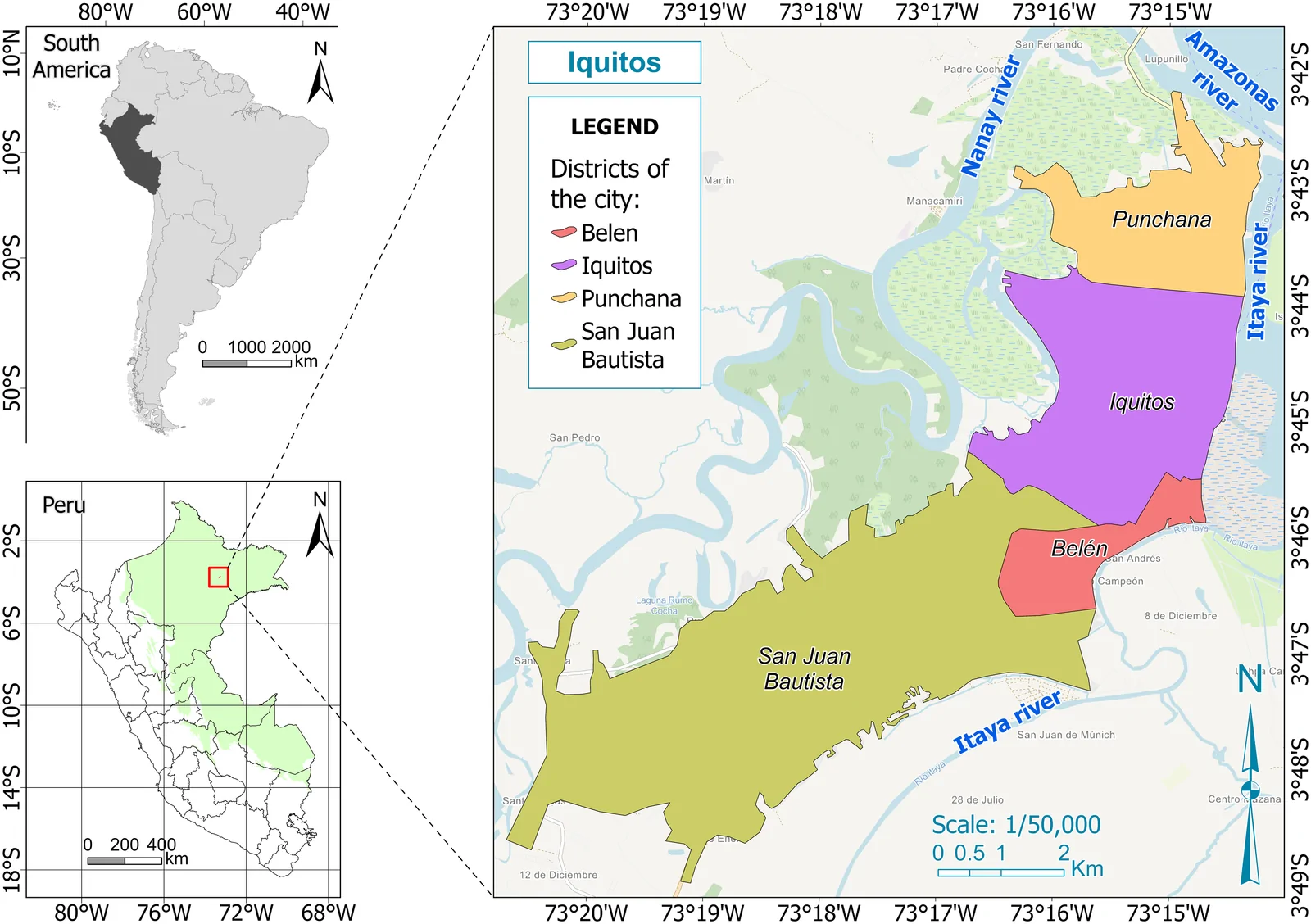
Geographic location of the study site in Iquitos city, Loreto Region, Peruvian Amazon. The map shows the four urban districts: Iquitos, San Juan Bautista, Punchana, and Belén. South America inset: country borders from geoBoundaries, an open global database of political-administrative boundaries (https://www.geoboundaries.org). Peru inset: ecoregions layer from MINAM, 2017 (https://geoservidor.minam.gob.pe/informacion-institucional/intercambio-de-datos/); departmental borders from GeoSDOT (https://geosdot.servicios.gob.pe/visor/). Iquitos map: district boundaries from INEI, 2023 (https://geosdot.servicios.gob.pe/visor/); base map from OpenStreetMap (OpenStreetMap contributors, ODbL; https://www.openstreetmap.org/copyright).

Previous studies in the Peruvian Amazon have consistently reported high *T. gondii* seroprevalence. A study in Iquitos city hospitals documented seroprevalence exceeding 97% in pregnant women [10], and more recent investigations across other Amazonian populations have confirmed very high exposure rates, highlighting the role of environmental, behavioral, and socioeconomic factors in sustaining transmission [11]. Environmental evidence further supports widespread oocyst contamination, with anti-*T. gondii* antibodies detected in Amazonian manatees (*Trichechus inunguis*) in the Loreto region [12], and a wide diversity of genotypes, including atypical highly virulent strains exhibiting a genetic diversity far greater than that observed in North America or Europe, identified in local wildlife [13]. Despite this evidence, comprehensive population-based seroprevalence data for the general urban population of Iquitos remain lacking, limiting a full characterization of infection burden and associated risk factors.

Therefore, this study aimed to determine the seroprevalence of anti-*T. gondii* IgG and IgM antibodies and to identify sociodemographic and behavioral factors associated with seropositivity in the urban population of Iquitos city, Peruvian Amazon. These findings will provide evidence-based epidemiological data to support the development of targeted prevention strategies for toxoplasmosis in this neglected tropical disease-endemic region.

## Materials and methods

### Ethics statement

This study was approved by the Institutional Review Board (IRB) of the Regional Hospital of Loreto (FWA00028456), through the Office of Support for Teaching and Research, in accordance with the guidelines of the Peruvian National Institute of Health (INS). The study was reviewed and approved following assessment of regional research priorities, risk–benefit analysis, data reliability, and ethical compliance, and was classified as a no-risk clinical study (Approval ID: 059-CIEI-HRL-2024). Written informed consent was obtained from all participants prior to their inclusion in the study. The study was conducted in adherence to the principles outlined in the Declaration of Helsinki.

### Study design, area, and population

A cross-sectional seroprevalence study was conducted from 11/07/2024 to 10/08/2024 in Iquitos city, the capital of the Loreto Region, located in the Peruvian Amazon basin (3°44′S, 73°15′W; elevation ~106 m a.s.l.). Iquitos is the largest city in the world inaccessible by road, characterized by a tropical rainforest climate (Köppen Af) with a mean annual temperature of 24–32°C, relative humidity consistently above 85%, and pronounced seasonal flooding that submerges large peri-urban areas for approximately six months per year, typically from December to May [8]. Participants were enrolled from the four administrative districts of the city: Iquitos, Punchana, Belén, and San Juan Bautista.

### Sample size and sampling strategy

To determine the sample size, we applied the formula for finite populations, assuming 95% confidence level, a 5% margin of error, and a conservative expected prevalence of 50% based on the population of Iquitos city (N = 422,055). Under these parameters, the minimum required sample size was calculated to be approximately 384 individuals.

To ensure representativeness, a simple random probability sampling method was applied. Households within the four districts were randomly selected from updated local records, and all eligible residents had the same likelihood of inclusion until the required sample size was reached. Eligible participants were those aged 18 years or older who had resided in Iquitos city for at least six continuous months, provided informed consent, and were present during the household visit. Exclusion criteria were refusal to participate, age under 18 years, residence outside of Iquitos city, and inability to communicate in writing. We excluded two individuals from the analysis due to incomplete epidemiological records, resulting in a final sample of 382 participants.

Following the initial serological screening by IgG ELISA, all 358 IgG-seropositive participants were tested for IgM antibodies. IgM testing was restricted to IgG-positive individuals, IgM + /IgG– profiles are considered extremely rare in population-based studies and are most likely attributable to false-positive IgM results or early seroconversion windows rather than true acute infection [14]. This two-stage serological approach –first establishing IgG seroprevalence in the full sample, then screening for IgM in the seropositive subgroup, was designed to identify serological profiles, including IgM positivity, that may reflect *T. gondii* exposure. It should be noted that *T. gondii* specific IgM antibodies can persist for 12–18 months or longer following primary infection; therefore, a positive IgM result should be interpreted as an orientating serological marker rather than a definitive indicator of acute infection, and confirmation by IgG avidity testing or paired serology would be required to establish the timing of infection more precisely [15–17].

### Questionnaire and epidemiological data collection

A structured questionnaire was used to collect data on demographic characteristics, socioeconomic status, housing conditions, pet ownership, dietary habits, and exposures potentially relevant to *T. gondii* transmission pathways. The full list of variables collected, including response categories and variable types, is provided in S1 Table.

### Biological samples

Blood samples (6 mL) were collected from participants via venipuncture, drawn into red-top tubes to allow for serum separation from the blood clot. These samples were then transported under refrigeration with ice packs to the laboratory. On the same day as collection, samples were centrifuged for 15 minutes at 3,500 rpm. The supernatant serum was carefully transferred to a new tube and stored at −20°C until analysis.

### Laboratory serological tests for *Toxoplasma gondii*

#### Detection of *T. gondii*-specific IgG antibodies.

*Toxoplasma gondii*-specific IgG antibodies were detected using an in-house ELISA, previously validated against the Vircell commercial kit (considered the gold standard), with demonstrated high sensitivity (SE: 98%) and specificity (SP: 100%) [11]. The total tachyzoite lysate antigen (TLA) was obtained through culture of the *T. gondii* RH strain, a representative of type I, following the protocol detailed [18]. Nunc MaxiSorp 96-well ELISA plates (Nunc Nalgene, Rochester, NY) were prepared by overnight coating at 4°C with 100 µL of TLA at 1 µg/mL in Carbonate Bicarbonate Buffer (Sigma C3041). After blocking with PBS–Tween 20 (0.05%) + 5% milk, diluted serum (1:500, 100 µL/well) was added and incubated for 1 hour at 37°C. Horseradish peroxidase (HRP) goat anti-human IgG (1: 10,000 dilution of 0.1 mg/mL stock) (SeraCare Life Sciences, Milford, MA, USA) was then incubated under the same conditions. TMB substrate (100 µL/well) (Thermo Fisher Scientific, Waltham, MA) was added for 5 minutes in the dark, and the reaction was stopped with 50 µL of 2 M H_2_SO_4_. Washes with PBS-T (0.05%) followed each step. Optical densities (ODs) were measured at 450 nm using an ELISA plate reader (Molecular Devices, LLC, San Jose, CA). Each plate included the same standardized serum pools as controls: high-positive (OD > 1.500), low-positive (OD ≤ 0.700), and negative controls. The cut-off value was calculated individually for each plate as the mean OD of the negative controls plus three standard deviations. Although inter-plate variability is expected, the cut-off values showed minimal variation across plates, indicating high assay reproducibility. A blank control was also included to correct for background absorbance.

#### Detection of *T. gondii*-specific IgM antibodies.

*T. gondii*-specific IgM antibodies were detected using the commercial ELISA kit Anti-*Toxoplasma gondii* IgM Capture (Vircell S.L., Granada, Spain; catalog code M1027), following the manufacturer’s instructions. According to the manufacturer’s product insert (L-M1027-ES-02), the assay demonstrated a sensitivity of 98–100% and specificity of 97–100% across two independent validation studies.

### Statistical analysis

*Toxoplasma gondii* seroprevalence for IgG and IgM antibodies was estimated using a binomial exact test. To evaluate potential associations between *T. gondii* IgG seropositivity and categorical variables, bivariate analyses were conducted using a Poisson regression model with robust variance, and variables with a global p-value < 0.20 were considered for inclusion in a manual forward stepwise nested model to identify predictors of *T. gondii* seropositivity. The nested multivariable model was built using Poisson regression with robust variance, and variables were retained based on the Akaike Information Criterion (AIC). Multicollinearity among covariates in the final model was assessed using the Variance Inflation Factor (VIF). Adjusted prevalence ratios (aPRs) with 95% confidence intervals were reported for the final multivariate model. For IgM, due to the very low number of positive cases, only bivariate analyses using Fisher’s exact test were performed, and no regression-based modelling was conducted. All data were analyzed using Stata version 19 (StataCorp, College Station, TX, USA).

### Use of artificial intelligence tools

The authors used an AI-based language model (Claude, claude-sonnet-4–6, San Francisco, CA, USA) for language editing of the Discussion section. All AI-assisted content was reviewed, verified, and approved by the authors, who take full responsibility for the accuracy and integrity of the manuscript.

## Results

### Socio-demographic characteristics

A total of 382 participants from the four districts of Iquitos city were enrolled in this study. The majority were female (243, 63.6%). Participants ranged in age from 18 to 82 years, with adults aged 25–59 years representing the largest group (198, 51.8%), followed by youth aged 18–24 years (145, 38.0%), and older adults aged >60 years (39, 10.2%). Among female participants, nine (3.7%) were pregnant at the time of enrollment, of whom the majority were multigravida (7/9, 77.8%). Regarding monthly family income, the largest proportion of participants earned ≤371 USD (141/382, 36.9%). Participants were approximately evenly distributed across the four districts. Most participants resided in urban areas (293, 76.7%), and approximately half had completed or were pursuing university education (183, 48.1%). The majority lived in houses constructed with brick, cement, or tile (286, 74.9%), and nearly all had access to electricity (371, 97.1%). For drinking water, more than half reported consuming treated or boiled water (223, 58.4%). The full sociodemographic and serological distribution is presented in Table 1.

**Table 1:** Sociodemographic characteristics and *Toxoplasma gondii* IgG seroprevalence in 382 adults from Iquitos, Peru, 2024.

Characteristics		N (%)	IgG Seropositive n (%)
**Sex**			
	Male	139 (36.4)	127 (91.4)
	Female	243 (63.6)	231 (95.0)
**Age group (years)**			
	Youth (18–24)	145 (38.0)	130 (89.7)
	Adult (25–59)	198 (51.8)	189 (95.5)
	Older adult (>60)	39 (10.2)	39 (100.0)
**Civil status**			
	Single/Widowed	235 (61.5)	215 (91.5)
	Married/Cohabiting	147 (38.5)	143 (97.3)
**Monthly family income (USD)**			
	≤371	141 (36.9)	133 (94.3)
			
	372–593	103 (27.0)	100 (97.1)
	594–1039	80 (20.9)	77 (96.3)
	>1040	58 (15.2)	48 (82.8)
**District**			
	Iquitos	96 (25.1)	91 (94.8)
	Punchana	91 (23.8)	81 (89.0)
	Belén	97 (25.4)	95 (97.9)
	San Juan Bautista	98 (25.7)	91 (92.9)
**Residential zone**			
	Urban	293 (76.7)	270 (92.1)
	Peri-urban/Rural	89 (23.3)	88 (98.9)
**Education level †**			
	Primary school	45 (11.8)	45 (100.0)
	High school	111 (29.1)	104 (93.7)
	Technical school	42 (11.0)	40 (95.2)
	University	183 (48.1)	168 (91.8)
**House construction material**			
	Wood	96 (25.1)	95 (99.0)
	Brick/cement/tile	286 (74.9)	263 (92.0)
**Drinking water source**			
	Tap water	141 (36.9)	131 (92.9)
	Treated/boiled water	223 (58.4)	209 (93.7)
	Other (river/rain)	18 (4.7)	18 (100.0)
**ELISA IgG result**			
	Seronegative	24 (6.3)	—
	Seropositive	358 (93.7)	358 (100)

† Some variables may total less than 382 due to missing data. Income categories expressed in USD; original values in Peruvian sol (PEN) converted at an approximate exchange rate of 1 USD = 3.37 PEN (2024 average).

### *Toxoplasma gondii* seroprevalence

The overall seroprevalence of *T. gondii* IgG antibodies was 93.7% (358/382; 95% CI: 90.8–95.9) (Fig 2). The scatter plot of optical density (OD) values at 450 nm showed a clear bimodal distribution, with the established cut-off at OD = 0.320. Among seropositive individuals, OD values clustered predominantly in the moderate range (0.701–1.500), accounting for 74.6% (267/358) of seropositive samples, followed by low OD (0.321–0.700; 18.7%, n = 67) and high OD (>1.500; 6.7%, n = 24). The coefficient of variation was markedly higher among seronegative individuals (59.0%) than positive ones (32.2%), reflecting greater relative variability at low antibody levels.

**Fig 2:**
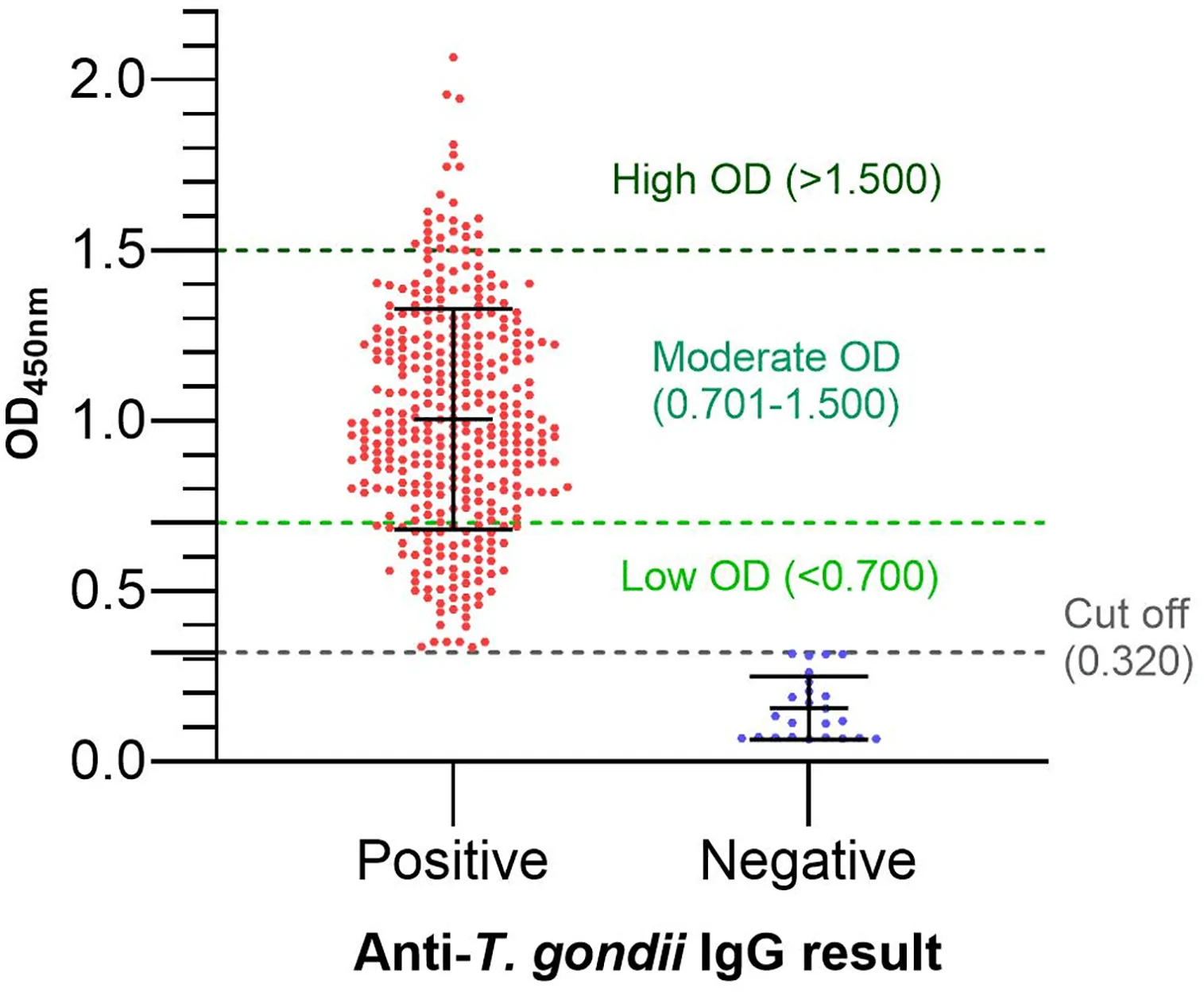
Distribution of anti-*Toxoplasma gondii* IgG optical density values in 382 serum samples from Iquitos city, Peru. The dashed line indicates the cut-off value (OD = 0.320). Seronegative: n = 24 (6.3%); Seropositive: n = 358 (93.7%).

### Factors associated with *Toxoplasma gondii* seropositivity IgG seropositivity

Bivariate analysis identified several factors associated with *T. gondii* IgG seropositivity (Table 2). Seropositivity increased with age: older adults (>60 years) had a 12% higher prevalence than youth aged 18–24 years (PR: 1.12; 95% CI: 1.06–1.18). Pregnant women also had higher seropositivity than non-pregnant women (PR: 1.05; 95% CI: 1.02–1.09). Peri-urban/rural residents had a 7% higher prevalence than urban residents (PR: 1.07; 95% CI: 1.03–1.12), and participants living in wooden housing had higher seropositivity than those in brick or cement houses (PR: 0.93; 95% CI: 0.89–0.97). Use of river or rainwater as a drinking water source was associated with higher seropositivity (PR: 1.08; 95% CI: 1.03–1.13), whereas higher education level, access to electricity, presence of a cat litter box inside the home, and consumption of processed meat or artisanal cheese were inversely associated with seropositivity. Variables with no significant association, including sex, district of residence, animal ownership, and most dietary variables are presented in S2 Table.

**Table 2:** Bivariate and multivariate analysis of factors associated with *Toxoplasma gondii* IgG seropositivity in adults from Iquitos, Peru (n = 382).

Characteristics	N (%)	IgG Neg N (%)	IgG Pos N (%)	PR (CI)	p-value	aPR (CI)**	p-value
Age (numerical)*	35.4 (± 16.8)	26.2 (± 9.6)	36.1 (± 17.0)	1.02 (1.00-1.03)	**<0.001**	1.05 (1.01-1.09)	**0.013**
Group age: Adult (25–59)	198 (51.8)	9 (4.6)	189 (95.5)	1.06 (0.99-1.13)	**0.052**	1.06 (0.99-1.13)	0.099
Group age: Older Adult (>60)	39 (10.2)	0 (0.0)	39 (100.0)	1.12 (1.06-1.18)	**<0.001**	1.10 (1.04-1.17)	**0.002**
Civil status: Married/Cohabiting	147 (38.5)	4 (2.7)	143 (97.3)	1.06 (1.01-1.12)	**0.011**	1.03 (0.99-1.08)	0.172
Pregnant women	9 (3.7)	0 (0.0)	9 (100.0)	1.05 (1.02-1.09)	**0.001**	1.03 (0.99-1.07)	0.117
Monthly family income (USD): 372–593	103 (27.0)	3 (2.9)	100 (97.1)	1.03 (0.98-1.08)	0.282	1.07 (1.01-1.15)	**0.035**
Monthly family income (USD): > 1040	58 (15.2)	10 (17.2)	48 (82.8)	0.88 (0.77-0.99)	**0.039**	0.92 (0.80-1.05)	0.248
Residence zone: Peri-urban/ Rural	89 (23.3.)	1 (1.1)	88 (98.9)	1.07 (1.03 -1.12)	**0.001**	1.04 (1.01-1.08)	**0.025**
Education Level: High school	111 (29.1)	7 (6.3)	104 (93.7)	0.94 (0.89-0.98)	**0.008**	0.99 (0.94-1.04)	0.778
Education level: University	183 (48.1)	15 (8.2)	168 (91.8)	0.92 (0.87-0.96)	**<0.001**	1.02 (0.95-1.10)	0.583
House: material Brick/cement/tile	286 (74.9)	23 (8.0)	263 (92.0)	0.93 (0.89-0.97)	**<0.001**	0.95 (0.91-0.99)	**0.037**
Electricity services: Yes	371 (97.1)	24 (6.5)	347 (93.5)	0.94 (0.91-0.96)	**<0.001**	1.02 (0.96-1.08)	0.595
Drinking water source: river/rain	18 (4.7)	0 (0.0)	18 (100.0)	1.08 (1.03-1.13)	**0.002**	1.08 (1.02-1.14)	**0.010**
Processed/cured meat: Yes	226 (59.8)	20 (8.9)	206 (91.1)	0.94 (0.89-0.98)	**0.007**	0.58 (0.18-1.91)	0.372
Fresh/artisanal cheese Yes	244 (64.6)	20 (8.2)	224 (91.8)	0.95 (0.90-0.99)	**0.024**	0.97 (0.92-1.02)	0. 174

*Media (SD), ** Adjusted for age, monthly family income, house construction material, drinking water source, fresh/artisanal cheese consumption, electricity services, and residential zone. Bold values indicate p < 0.05. PR: Prevalence ratio; aPR: adjusted PR; CI: confidence interval. Only significant variables shown; full data in S2 Table.

### Multivariable analysis of factors associated with *Toxoplasma gondii* IgG

In the final multivariable model, associations with *T. gondii* seropositivity were adjusted for age, monthly family income, house construction material, drinking water source, fresh/artisanal cheese consumption, electricity services, and residential zone. Age remained an independent predictor of *T. gondii* seropositivity after adjustment. When modelled as a continuous variable, each additional year of age was associated with a 5% higher prevalence of seropositivity (aPR: 1.05; 95% CI: 1.01–1.09). By age group, older adults (>60 years) had 10% higher seroprevalence than youth aged 18–24 years (aPR: 1.10; 95% CI: 1.04–1.17), whereas the association among adults aged 25–59 years was attenuated after adjustment (aPR: 1.05; 95% CI: 0.98–1.12).

Monthly family income remained associated with seropositivity. Participants with a monthly family income of 372–593 USD had 7% higher seroprevalence than those in the lowest income category (aPR: 1.07; 95% CI: 1.01–1.15), whereas the lower seroprevalence observed among participants in the highest income category (>USD 1,040) was attenuated after adjustment (aPR: 0.92; 95% CI: 0.80–1.05).

Peri-urban/rural residence was independently associated with slightly higher seropositivity after adjustment, with residents exhibiting 4% higher seroprevalence than urban participants (aPR: 1.04; 95% CI: 1.01–1.08). Housing characteristics also remained associated with seropositivity after adjustment; participants living in houses with brick, cement, or tile had 5% lower seroprevalence than those living in wooden houses (aPR: 0.95; 95% CI: 0.91–0.99). Additionally, use of river or rainwater as a drinking water source was associated with 8% higher seroprevalence compared with treated or bottled water after adjustment (aPR: 1.08; 95% CI: 1.02–1.14).

Several factors associated with seropositivity in bivariate analyses, including civil status, education level, presence of a cat litter box, and consumption of processed/cured meat and fresh/artisanal cheese, were attenuated after adjustment and were no longer independently associated with seropositivity.

### IgM seropositivity

IgM antibodies were assessed exclusively in the 358 IgG-seropositive participants (i.e., not in the 24 IgG-seronegative individuals); sociodemographic and exposure characteristics of this subgroup are reflected in Table 1. The overall IgM seroprevalence was 1.7% (6/358; 95% CI: 0.62-3.6%). Due to the low number of IgM-positive cases, analyses were restricted to bivariate comparisons using Fisher’s exact test (Table 3). Monthly family income was associated with IgM seropositivity, with higher proportions observed among participants in the USD 594–1,039 (3.9%) and>USD 1,040 (4.2%) income categories compared with lower-income groups (p = 0.043). Similarly, participants who did not consume fresh or artisanal cheese had higher IgM seropositivity than consumers (3.1% vs. 0.5%, p = 0.043). Non-consumers of red meat also had higher IgM seropositivity than consumers (6.3% vs. 1.2%, p = 0.035).

**Table 3:** Bivariate analysis of factors associated with *Toxoplasma gondii* IgM seropositivity in adults from Iquitos, Peru (n = 358).

Characteristics	N (%)	IgM Neg. N (%)	IgM Pos. N (%)	p-value
**Monthly family income (USD)**				**0.043**
≤371	133 (37.2)	132 (99.3)	1 (0.7)	
372–593	100 (27.9)	100 (100.0)	0 (0.0)	
594–1039	77 (21.5)	74 (96.1)	3 (3.9)	
>1040	48 (13.4)	46 (95.8)	2 (4.2)	
**Cat litter box inside home**				0.085
No	281 (78.5)	276 (98.2)	5 (1.8)	
Yes	77 (21.5)	76 (98.7)	1 (1.3)	
**Red meat consumption**				**0.035**
No	32 (8.9)	30 (93.7)	2 (6.3)	
Yes	326 (91.1)	322 (98.8)	4 (1.2)	
**Fresh/artisanal cheese†**				**0.043**
No	130 (36.7)	126 (96.9)	4 (3.1)	
Yes	224 (63.3)	223 (99.5)	1 (0.5)	

† Missing data. All analyses by Fisher’s exact test. Only significant and near-significant variables shown; full data in S3 Table.

The presence of a cat litter box inside the home was not significantly associated with IgM seropositivity; IgM positivity was 1.3% among participants with a cat litter box compared with 1.8% among those without (p = 0.085). Full bivariate results for IgM seropositivity are provided in S3 Table.

## Discussion

We found a very high IgG seroprevalence against *T. gondii* (93.7%) in the urban population of Iquitos. This rate is among the highest described for any general urban population worldwide. It greatly exceeds global estimates [19] and even many tropical settings [2,3] and is consistent with prior reports in pregnant women from the same city (97.4–97.6%) [10]. These findings are further corroborated by other Peruvian Amazon studies: Silva-Díaz and Díaz-Ginéz [20] reported 75.3% seroprevalence among blood donors in Tarapoto, and Menajovsky et al. [21] documented high exposure (83.3% IgG and 6.1% IgM) among indigenous communities in Loreto. Collectively, these data place the Peruvian Amazon among the most endemic regions for toxoplasmosis globally and highlight the need for targeted public health action in this setting.

The high seroprevalence in this region is not surprising given the local environmental conditions. The Amazon basin has high temperatures and humidity year-round, which promote oocyst sporulation and extend viability in soil and water [22,23], abundant domestic and feral feline populations, widespread reliance on untreated water sources, and dietary practices common in the region. It should be noted that, in this study, no dietary variable showed a significant independent association with *T. gondii* seropositivity in multivariable analysis; the mention of dietary practices here refers to the broader ecological context rather than to a specific measured dietary risk factor in this population. *T. gondii* antibodies have been detected in Amazonian manatees (*Trichechus inunguis*) in Loreto’s waterways [5,12]. Gennari et al. [13] identified atypical and recombinant *T. gondii* genotypes circulating in the Amazon region, reflecting a far more diverse parasite population than that observed in temperate regions such as North America or Europe. This exceptionally high diversity may also contribute to the high cumulative seroprevalence.

Age was strongly associated with seropositivity. Rates increased from 89.7% in the 18–24 years’ group to 100% in adults over 60, which reflects the cumulative effect of prolonged exposure in an endemic setting [3,24]. The already high seroprevalence in young adults suggests that infection is typically acquired early in life. Future studies should include children to better understand at what age transmission risk is highest.

Income and type of housing material were associated with seropositivity in bivariate analysis. This is consistent with reports from other Amazonian populations [21,25,26]. After adjustment, both associations were attenuated considerably. These variables may be proxies for socioeconomic status or more direct unmeasured environmental exposures such as limited access to safe water and sanitation [27]. The independent associations with wooden housing and river/rainwater use point to structural poverty and inadequate infrastructure as primary drivers of transmission in peri-urban Iquitos.

Two variables in the multivariable model deserve specific comment. The use of river or rainwater for drinking was independently associated with 8% higher seroprevalence (aPR: 1.08; 95% CI: 1.02–1.14; p < 0.01). This was the only modifiable environmental exposure that remained significant in the adjusted model. It provides epidemiological evidence for waterborne transmission in Iquitos, consistent with detection of oocyst contamination in Loreto’s water bodies [12]. In several districts of the city, municipal water supply is not continuous. Many families respond by storing water in open containers or using surface water, practices that increase the chance of oocyst ingestion [4]. Expanding access to treated water in peri-urban areas is therefore a priority intervention. Wooden housing was the other independent predictor. These structures are very common in flood-prone areas like the Belén district. They tend to have earthen floors, insufficient waste management, and are more permeable to rodents and insects, all conditions that support oocyst persistence [5,28]. While wooden housing is likely not causally linked to *T. gondii* exposure, it likely serves as a marker of increased environmental exposure to the parasite. Together, these findings point to infrastructure deficits rather than individual behaviors as the main drivers of infection in peri-urban Iquitos.

We also observed inverse associations between seropositivity and cat litter box use, processed meat consumption, and artisanal cheese intake. These results are probably explained by socioeconomic confounding. Families that can afford litter boxes and commercial food products generally have higher incomes, better housing conditions, and improved access to potable water and sanitation [29–32]. Having an indoor litter box also likely reflects keeping cats inside the home, which may reduce feline contact with infected prey animals and thus limit *T. gondii* oocyst shedding in the household, further reducing household exposure.

The IgM seroprevalence of 1.7% (6/358) among IgG-positive individuals is low but not negligible, and it is well below the approximately 10% IgM positivity previously reported in pregnant women from Iquitos [10]. This difference probably reflects the broader age range of our sample, since older IgG-positive adults were almost certainly infected in childhood or early adulthood rather than recently. We also may have underestimated the true IgM rate slightly, given that we tested only IgG-positive individuals and therefore missed any very early infections occurring before IgG seroconversion. The counterintuitive association of IgM seropositivity with higher income brackets warrants caution in interpretation and may be attributable in part to residual confounding or to the known prolonged persistence of *T. gondii*-specific IgM antibodies, which can remain detectable for 12–18 months or longer after primary infection and therefore do not reliably indicate acute or recent infection [14–17]. An alternative explanation is that, in lower-income households where childhood infection is virtually universal, adults have long since acquired immunity and are no longer susceptible to primary infection, whereas higher-income individuals who avoided early exposure may still be at risk of acquiring *Toxoplasma* for the first time. In either case, a positive IgM result in an IgG-positive individual should be interpreted as an orientating serological marker only; confirmation by IgG avidity testing or paired serology would be required to establish the timing of infection [14,15].

The public health implications of these findings are clear. Children, younger adults, and peri-urban residents should be prioritized in surveillance and prevention programs. Because seroprevalence is already near-universal in adults, individual-level behavioral interventions are unlikely to have much impact. Investment should instead go toward improving water treatment infrastructure, sanitation, and housing conditions in peri-urban areas [33]. Multisectoral One Health approaches integrating community participation, feline population management, and environmental sanitation are essential for sustainable impact in this complex Amazonian setting [7,34].

Given the very high prevalence of *T. gondii* infection in this population, certain high-risk patient groups should receive particular focus. Routine serological screening of pregnant women for IgG and IgM antibodies should be a public health priority, given the risk of congenital toxoplasmosis [6,10]. Additionally, near-universal *T. gondii* seroprevalence has critical implications for people living with HIV in Loreto, a region with the highest HIV-associated mortality in Peru: virtually every person with advanced HIV is at risk of toxoplasmic encephalitis through reactivation of latent infection, as documented by Steinberg et al. [35], who identified cerebral toxoplasmosis in 24% of HIV patients with acute neurological symptoms at Hospital Regional de Loreto. Integrating routine *T. gondii* screening and primary prophylaxis into HIV care programs, particularly for individuals with CD4 counts below 100 cells/mm^3^, is therefore a priority.

This study has several limitations. The cross-sectional design prevents causal inference. The near-universal seroprevalence also created a ceiling effect that limited statistical power and probably explains some of the unexpected inverse associations in bivariate analysis. Most participants were infected years or decades before the study, so their current dietary habits and behaviors may not reflect their actual exposures at the time of infection. Self-reported dietary data are also subject to recall bias. The study was limited to adults in one city, so findings cannot be generalized to children, rural populations, or other regions. We excluded children mainly for ethical and practical reasons related to venipuncture, but this means we cannot determine at what age infection risk is greatest, which should be addressed in future studies using alternative sampling approaches, such as fingerprick collection or dried blood spot testing, which are routinely used in pediatric populations. Finally, some relevant exposure variables were not collected, including rodent presence near the home, specific cat-related data, and history of prior parasitic infections.

In conclusion, *T. gondii* seroprevalence in the adult population of Iquitos is very high, reaching near-universal levels in older age groups. Age, income bracket, and peri-urban or rural residence were independent predictors of seropositivity. Many associations seen in bivariate analysis were explained by socioeconomic confounding after adjustment. These results support the need for stronger toxoplasmosis surveillance, routine antenatal serological screening, and, most importantly, investments in water treatment and sanitation in peri-urban areas of the Peruvian Amazon. Reducing the burden of this neglected disease in the region will require intersectoral coordination and approaches adapted to local environmental and social conditions.

## Supporting information

S1 Table Variables collected in the epidemiological questionnaire administered to study participants in Iquitos, Loreto, Peru, 2024. The table lists all epidemiological variables collected, organized by domain (sociodemographic characteristics, housing conditions, pet ownership and animal exposure, hygiene habits, dietary habits, knowledge about toxoplasmosis, medical history, and current symptoms), along with response categories and variable type.
(DOCX)

S2 Table Bivariate analysis of all sociodemographic and exposure variables not associated with *Toxoplasma gondii* IgG seropositivity in 382 adults from Iquitos city, Peru, 2024. Variables with p > 0.05 are shown.
(DOCX)

S3 Table Bivariate analysis of all sociodemographic and exposure variables not associated with *Toxoplasma gondii* IgM seropositivity in 358 IgG-seropositive adults from Iquitos city, Peru, 2024. Variables with p > 0.05 are shown. All analyses were performed using Fisher’s exact test.
(DOCX)
