# Supplementary material for: High seroprevalence and risk factors for Toxoplasma gondii infection in Iquitos: A city in the Peruvian Amazon
Source: PLoS Negl Trop Dis. 2026 Nov 10;20(11):e0014476. doi: 10.1371/journal.pntd.0014476 (PMC13766600; doi:10.1371/journal.pntd.0014476)
Supplement: Supplementary file 1 [file pntd.0014476.s001.docx]

**S1 Table. Variables collected in the epidemiological questionnaire administered to study participants in Iquitos, Loreto, Peru, 2024.**

| **Variable** | **Response categories / Description** | **Type** |
| --- | --- | --- |
| **1. Sociodemographic characteristics** | | |
| Age | Continuous (years) | Continuous |
| Sex | Male / Female | Categorical |
| Pregnancy status (women only) | Yes / No | Categorical |
| Gravidity (if pregnant) | Primigravida / Multigravida | Categorical |
| Marital status | Single / Married / Cohabiting / Widowed | Categorical |
| Monthly family income | Continuous (PEN); categorized as ≤1,250 / 1,251–2,000 / 2,001–3,500 / >3,500 PEN. For presentation purposes, income categories were converted from Peruvian soles (PEN) to U.S. dollars (USD) ≤$371; $372–593; $594–1,039; >$1,040 | Continuous / Ordinal |
| District of residence | Iquitos / Punchana / Belén / San Juan Bautista | Categorical |
| Residential zone | Urban / Peri-urban or marginal urban / Rural | Categorical |
| Education level | Primary / Secondary / Technical / University (complete or incomplete) | Ordinal |
| Occupation | Student / Self-employed / Employed / Unemployed / Homemaker / Other | Categorical |
| Religion | Catholic / Evangelical / Other | Categorical |
| **2. Housing conditions** | | |
| House construction material | Wood / Brick, cement, or tile / Other | Categorical |
| Access to electricity | Never / Sometimes / Almost always / Always | Ordinal |
| Primary drinking water source | Public network / Treated water / Boiled water / Well water / Other | Categorical |
| **3. Pet ownership and animal exposure** | | |
| Cat ownership | Yes / No | Categorical |
| Number of cats (if owner) | Continuous | Continuous |
| Cat access to outdoors | Yes / No | Categorical |
| Presence of cat litter box inside home | Yes / No | Categorical |
| Litter box cleaning frequency | Daily / Every 2–3 days / Weekly / Less frequently | Ordinal |
| Ownership of other domestic animals | Yes / No; type specified | Categorical |
| **4. Hygiene habits** | | |
| Handwashing before eating | Always / Almost always / Sometimes / Never | Ordinal |
| Handwashing after handling soil or sand | Always / Almost always / Sometimes / Never | Ordinal |
| Gardening activities | Yes / No | Categorical |
| Use of gloves when handling soil | Always / Sometimes / Never | Ordinal |
| **5. Dietary habits** | | |
| Meat consumption | Yes / No | Categorical |
| Meat consumption frequency | Daily / 2–3 times per week / Weekly / Less frequently | Ordinal |
| Usual meat cooking level | Well done / Medium / Rare / Raw | Ordinal |
| Processed or cured meat consumption | Yes / No | Categorical |
| Raw vegetable (salad) consumption | Yes / No | Categorical |
| Fruit consumption | Yes / No | Categorical |
| Fruit and vegetable washing method | Tap water / Bottled water / Well water / Disinfectant solution | Categorical |
| Home garden produce consumption | Yes / No | Categorical |
| Milk consumption | Yes / No | Categorical |
| Type of milk consumed | Pasteurized / UHT / Unpasteurized (raw) | Categorical |
| Fresh or artisanal cheese consumption | Yes / No | Categorical |
| **6. Knowledge about toxoplasmosis** | | |
| Awareness of toxoplasmosis | Yes / No | Categorical |
| Knowledge of transmission routes | Yes / No | Categorical |
| Knowledge of symptoms | Yes / No | Categorical |
| Knowledge of risks during pregnancy | Yes / No | Categorical |
| **7. Medical history** | | |
| Previous toxoplasmosis diagnosis | Yes / No | Categorical |
| Blood transfusion in past 5 years | Yes / No | Categorical |
| Immunocompromising condition | Yes (HIV / Cancer / Organ transplant / Other) / No | Categorical |
| Prior serological screening during pregnancy (women) | Yes / No / Not applicable | Categorical |
| **8. Current symptoms (past few weeks)** | | |
| Fever | Yes / No | Categorical |
| Fatigue | Yes / No | Categorical |
| Muscle pain | Yes / No | Categorical |
| Lymph node swelling | Yes / No | Categorical |

*PEN: Peruvian sol. Income categories were originally defined in PEN and converted to U.S. dollars (USD) for presentation using an approximate exchange rate of 1 USD = 3.37 PEN (2024 average). Variables not significantly associated with T. gondii IgG or IgM seropositivity in bivariate analysis are reported in S2 Table and S3 Table, respectively.*
