# Supplementary material for: High seroprevalence and risk factors for Toxoplasma gondii infection in Iquitos: A city in the Peruvian Amazon
Source: PLoS Negl Trop Dis. 2026 Nov 10;20(11):e0014476. doi: 10.1371/journal.pntd.0014476 (PMC13766600; doi:10.1371/journal.pntd.0014476)
Supplement: Supplementary file 2 [file pntd.0014476.s002.docx]

**S2 Table. Sociodemographic and exposure variables non-associated with *Toxoplasma gondii* IgG seropositivity in 382 IgG-seropositive adults from Iquitos, Peru, 2024.**

| **Characteristics** | **N (%)** | **IgG Neg  N (%)** | **IgG Pos N (%)** | **PR (CI)** | **p-value** | **aPR (CI)**** | **p-value** |  |
| --- | --- | --- | --- | --- | --- | --- | --- | --- |
| **Sex** |  |  |  |  |  |  |  |  |
| Male | 139 (36.4) | 12 (8.6) | 127 (91.4) | Ref. | **-** | **Ref** | **-** |  |
| Female | 243 (63.6) | 14 (5.0) | 231 (95.0) | 1.04 (0.98-1.10) | 0.185 | 1.03 (0.97-1.09) | 0.315 |  |
| **Age (numerical)*** | 35.4 (± 16.8) | 26.2 (± 9.6) | 36.1 (± 0.9) | 1.02 (1.00-1.03) | **<0.001** | 1.05 (1.01-1.09) | **0.013** |  |
| **Age group (years)** |  |  |  |  |  |  |  |  |
| Youth (18-24) | 145 (38.0) | 15 (10.3) | 130 (89.7) | Ref. | **-** | Ref. | - |  |
| Adult (25-59) | 198 (51.8) | 9 (4.6) | 189 (95.5) | 1.06 (0.99-1.13) | **0.052** | 1.06 (0.99-1.13) | 0.099 |  |
| Older Adult (>60) | 39 (10.2) | 0 (0.0) | 39 (100.0) | 1.12 (1.06-1.18) | **<0.001** | **1.10 (1.04-1.17)** | **0.002** |  |
| **Civil status** |  |  |  |  |  |  |  |  |
| Single/Widowed | 235 (61.5) | 20 (8.5) | 215 (91.5) | Ref. |  | Ref. |  |  |
| Married/Cohabiting | 147 (38.5) | 4 (2.7) | 143 (97.3) | 1.06 (1.01-1.12) | **0.011** | 1.03 (0.99-1.08) | 0.172 |  |
| **Pregnancy status †** |  |  |  |  |  |  |  |  |
| Not pregnant | 234 (96.3) | 12 (5.1) | 222 (94.9) | Ref. | **-** | Ref | **-** |  |
| Pregnant | 9 (3.7) | 0 (0.0) | 9 (100.0) | 1.05 (1.02-1.09) | **0.001** | 1.03 (0.99-1.07) | 0.117 |  |
| **Monthly family income (PEN)** |  |  |  |  |  |  |  |  |
| ≤1,250 | 141 (36.9) | 8 (5.7) | 133 (94.3) | Ref. | - | Ref. | - |  |
| 1,251–2,000 | 103 (27.0) | 3 (2.9) | 100 (97.1) | 1.03 (0.98-1.08) | 0.282 | 1.07 (1.01-1.15) | **0.035** |  |
| 2,001–3,500 | 80 (20.9) | 3 (3.8) | 77 (96.3) | 1.02 (0.96-1.08) | 0.505 | 1.07 (0.99-1.15) | 0.073 |  |
| >3,500 | 58 (15.2) | 10 (17.2) | 48 (82.8) | 0.88 (0.77-0.99) | **0.039** | 0.92 (0.80-1.05) | 0.248 |  |
| **District** |  |  |  |  |  |  |  |  |
| Iquitos | 96 (25.1) | 5 (5.2) | 91 (94.8) | Ref. | **-** | Ref. | **-** |  |
| Punchana | 91 (23.8) | 10 (11.0) | 81 (89.0) | 0.94 (0.86-1.02) | 0.153 | 0.96 (0.88-1.04) | 0.314 |  |
| Belen | 97 (25.4) | 2 (2.1) | 95 (97.9) | 1.03 (0.97-1.09) | 0.246 | 1.03 (0.96-1.09) | 0.482 |  |
| San Juan | 98 (25.7) | 7 (7.1) | 91 (92.9) | 0.98 (0.91-1.05) | 0.576 | 0.98 (0.91-1.06) | 0.639 |  |
| **Residential Zone** |  |  |  |  |  |  |  |  |
| Urban | 293 (76.7) | 23 (7.9) | 270 (92.1) | Ref. | - | Ref. | - |  |
| Peri-urban/Rural | 89 (23.3.) | 1 (1.1) | 88 (98.9) | 1.07 (1.03 -1.12) | **0.001** | 1.04 (1.01-1.08) | **0.025** |  |
| **Education Level †** |  |  |  |  |  |  |  |  |
| Primary school | 45 (11.8) | 0 (0.0) | 45 (100.0) | Ref. | - | Ref. | - |  |
| High school | 111 (29.1) | 7 (6.3) | 104 (93.7) | 0.94 (0.89-0.98) | **0.008** | 0.99 (0.94-1.04) | 0.778 |  |
| Technical school | 42 (11.0) | 2 (4.8) | 40 (95.2) | 0.95 (0.89-1.02) | 0.158 | 1.04 (0.96-1.14) | 0.778 |  |
| University | 183 (48.1) | 15 (8.2) | 168 (91.8) | 0.92 (0.87-0.96) | **<0.001** | 1.02 (0.95-1.10) | 0.583 |  |
| **House construction material** |  |  |  |  |  |  |  |  |
| Wood | 96 (25.1) | 1 (1.0) | 95 (99.0) | Ref. | - | Ref. | - |  |
| Brick/cement/tile | 286 (74.9) | 23 (8.0) | 263 (92.0) | 0.93 (0.89-0.97) | **<0.001** | 0.95 (0.91-0.99) | **0.037** |  |
| **Electricity** |  |  |  |  |  |  |  |  |
| No | 11 (2.9) | 0 (0.0) | 11 (100.0 | Ref. | - | Ref. | _ |  |
| Yes | 371 (97.1) | 24 (6.5) | 347 (93.5) | 0.94 (0.91-0.96) | **<0.001** | 1.02 (0.96-1.08) | 0.595 |  |
| **Drinking water source** |  |  |  |  |  |  |  |  |
| Tap water | 141 (36.9) | 10 (7.1) | 131 (92.9) | Ref. | - | Ref. | - |  |
| Treated/boiled water | 223 (58.4) | 14 (6.3) | 209 (93.7) | 1.01 (0.95-1.07) | 0.764 | 1.03 (0.97-1.10) | 0.275 |  |
| Other (river/rain) | 18 (4.7) | 0 (0.0) | 18 (100.0) | 1.08 (1.03-1.13) | **0.002** | 1.08 (1.02-1.14) | **0.010** |  |
| **Cat inside home** |  |  |  |  |  |  |  |  |
| No | 177 (46.3) | 9 (5.1) | 168 (94.9) | Ref. | - | - | - |  |
| Yes | 205 (53.7) | 15 (7.3) | 190 (92.7) | 0.97 (0.93-1.03) | 0.365 | - | - |  |
| **Number of cats inside home** |  |  |  |  |  |  |  |  |
| None | 177 (46.3) | 9 (5.1) | 168 (94.9) | Ref. | - | - | - |  |
| One | 111 (29.1) | 9 (8.1) | 102 (91.9) | 0.97 (0.91-1.03) | 0.329 | - | - |  |
| Two or more | 44 (24.6) | 6 (6.4) | 88 (93.6) | 0.99 (0.93-1.05) | 0.668 | - | - |  |
| **Cat litter box inside home** |  |  |  |  |  |  |  |  |
| No | 295 (77.2) | 14 (4.8) | 281 (95.2) | Ref. | - | Ref. | - |  |
| Yes | 87 (22.8) | 10 (11.5) | 77 (88.5) | 0.93 (0.86-1.01) | 0.072 | 0.96 (0.89-1.04) | 0.301 |  |
| **Other animals inside home** |  |  |  |  |  |  |  |  |
| No | 217 (56.8) | 11 (5.1) | 206 (94.9) | Ref. | - | - | - |  |
| Yes | 165 (43.2) | 13 (7.9) | 152 (92.1) | 0.97 (0.91-1.02) | 0.278 | - | - |  |
| **Red meat consumption** |  |  |  |  |  |  |  |  |
| No | 33 (8.6) | 1 (3.0) | 32 (97.0) | Ref. | - | - | - |  |
| Yes | 349 (91.4) | 23 (6.6) | 326 (93.4) | 0.96 (0.90-1.03) | 0.270 | - | - |  |
| **Raw meat consumption** |  |  |  |  |  |  |  |  |
| No | 367 (96.1) | 23 (6.3) | 344 (93.7) | Ref. | - | - | - |  |
| Yes | 15 (3.9) | 1 (6.7) | 14 (93.3) | 0.99 (0.87-1.14) | 0.952 | - | - |  |
| **Processed/cured meat†** |  |  |  |  |  |  |  |  |
| Yes | 152 (40.2) | 4 (2.6) | 148 (97.4) | Ref. | - | Ref. | - |  |
| No | 226 (59.8) | 20 (8.9) | 206 (91.1) | 0.94 (0.89-0.98) | **>0.01** | 0.58 (0.18-1.91) | 0.372 |  |
| **Raw vegetables †** |  |  |  |  |  |  |  |  |
| No | 43 (11.4) | 4 (9.3) | 39 (90.7) | Ref. | - | - | - |  |
| Yes | 336 (88.6) | 20 (6.0) | 316 (94.0) | 1.03 (0.94-1.15) | 0.475 | - | - |  |
| **Homegrown produce consumption †** |  |  |  |  |  |  |  |  |
| No | 289 (77.1) | 18 (6.2) | 271 (93.8) | Ref. | - | - | - |  |
| Yes | 86 (22.9) | 6 (7.0) | 80 (93.0) | 0.99 (0.93-1.06) | 0.810 | - | - |  |
| **Fruit consumption †** |  |  |  |  |  |  |  |  |
| No | 30 (7.9) | 2 (6.7) | 28 (93.3) | Ref. | - | - | - |  |
| Yes | 350 (92.1) | 22 (6.3) | 328 (93.7) | 1.00 (0.90-1.11) | 0.936 | - | - |  |
| **Milk consumption †** |  |  |  |  |  |  |  |  |
| No | 77 (20.3) | 3 (3.9) | 74 (96.1) | Ref. | - | - | - |  |
| Yes | 303 (79.7) | 21 (6.9) | 282 (93.1) | 0.97 (0.92-1.02) | 0.249 | - | - |  |
| **Raw milk consumption** |  |  |  |  |  |  |  |  |
| No | 375 (98.2) | 23 (6.1) | 352 (93.8) | Ref. | - | - | - |  |
| Yes | 7 (1.8) | 1 (14.3) | 6 (85.7) | 0.91 (0.67-1.24) | 0.558 | - | - |  |
| **Fresh/artisanal cheese†** |  |  |  |  |  |  |  |  |
| No | 134 (35.5) | 4 (3.0) | 130 (97.0) | Ref. | - | Ref. | - |  |
| Yes | 244 (64.6) | 20 (8.2) | 224 (91.8) | 0.95 (0.90-0.99) | **0.024** | 0.97 (0.92-1.02) | 0. 174 |  |
| *Media (SD), ** Adjusted for age, monthly family income, house construction material, drinking water source, fresh/artisanal cheese consumption, electricity services, and residential zone. Bold values indicate p<0.05. PR: Prevalence ratio; aPR: adjusted PR; CI: confidence interval. | | | | | | | | |
