# Supplementary material for: High seroprevalence and risk factors for Toxoplasma gondii infection in Iquitos: A city in the Peruvian Amazon
Source: PLoS Negl Trop Dis. 2026 Nov 10;20(11):e0014476. doi: 10.1371/journal.pntd.0014476 (PMC13766600; doi:10.1371/journal.pntd.0014476)
Supplement: Supplementary file 3 [file pntd.0014476.s003.docx]

**S3 Table. Bivariate analysis of all sociodemographic and exposure variables non-associated with *Toxoplasma gondii* IgM seropositivity in 358 IgG-seropositive adults from Iquitos city, Peru, 2024.**

| **Characteristics** | **N (%)** | **IgG Neg  N (%)** | **IgG Pos N (%)** | **P-value** |
| --- | --- | --- | --- | --- |
| **Sex** |  |  |  | 0.429 |
| Male | 127 (35.5) | 126 (99.2) | 1 (0.8) |  |
| Female | 231 (64.5) | 226 (97.8) | 5 (2.2) |  |
| **Age (numerical)*** | 36.1 (±17.0) | 36.1 (±17.1) | 31.7 (± 11.4) | 0.524** |
| **Age group (years)** |  |  |  | 0.637 |
| Youth (18-24) | 130 (36.6) | 128 (98.5) | 2 (1.5) |  |
| Adult (25-59) | 189 (52.8) | 185 (97.9) | 4 (2.1) |  |
| Older Adult (>60) | 39 (10.9) | 39 (100) | 0 (0.0) |  |
| **Civil status** |  |  |  | 0.408 |
| Single/Widowed | 215 (60.1) | 210 (97.7) | 5 (2.3) |  |
| Married/Cohabiting | 143 (39.9) | 142 (99.3) | 1 (0.7) |  |
| **Pregnancy status †** |  |  |  | 0.182 |
| Not pregnant | 222 (96.1) | 218 (98.2) | 4 (1.8)) |  |
| Pregnant | 9 (3.9) | 8 (88.9) | 1 (11.1) |  |
| **District** |  |  |  | 0.299 |
| Iquitos | 91 (25.4) | 88 (96.7) | 3 (3.7) |  |
| Punchana | 81 (22.6) | 79 (97.5) | 2 (2.5) |  |
| Belen | 95 (26.5) | 94 (98.9) | 1 (1.1) |  |
| San Juan | 91 (25.5) | 91 (100) | 0 (0.0) |  |
| **Residential Zone** |  |  |  | 0.650 |
| Urban | 270 (75.4) | 265 (98.1) | 5 (1.9) |  |
| Peri-urban/Rural | 88 (24.6) | 87 (98.9) | 1 (1.1) |  |
| **Education Level †** |  |  |  | 0.232 |
| Primary school | 45 (12.6) | 44 (97.8) | 1 (2.2) |  |
| High school | 104 (29.1) | 104 (100) | 0 (0.0) |  |
| Technical school | 40 (11.2) | 40 (100) | 0 (0.0) |  |
| University | 168 (47.1) | 163 (97.0) | 5 (3.0) |  |
| **House construction material** |  |  |  | 0.497 |
| Wood | 95 (26.5) | 94 (98.9) | 1 (1.1) |  |
| Brick/cement/tile | 263 (73.5) | 258 (98.1) | 5 (1.9) |  |
| **Electricity** |  |  |  | 0.660 |
| No | 11 (3.1) | 11 (100) | 0 (0.0) |  |
| Yes | 347 (96.9) | 341 (98.3) | 6 (1.7) |  |
| **Drinking water source** |  |  |  | 0.820 |
| Tap water | 131 (36.6 ) | 129 (98.5) | 2 (1.5) |  |
| Treated/boiled water | 209 (58.4) | 205 (98.1) | 4 (1.9) |  |
| Other (river/rain) | 18 (5.0) | 18 (100.0) | 0 (0.0) |  |
| **Cat inside home** |  |  |  | 0.598 |
| No | 168 (46.9) | 165 (98.2) | 3 (1.8) |  |
| Yes | 190 (53.1) | 187 (98.4) | 3 (1.6) |  |
| **Number of cats inside home** |  |  |  | 0.897 |
| None | 168 (46.9) | 165 (98.2) | 3 (1.8) |  |
| One | 102 (28.5) | 100 (98.0) | 2 (2.0) |  |
| Two or more | 88 (24.6) | 87 (98.9) | 1 (1.1) |  |
| **Other animals inside home** |  |  |  | 0.493 |
| No | 206 (57.5) | 202 (98.1) | 4 (1.9) |  |
| Yes | 152 (42.5) | 150 (98.7) | 2 (1.3) |  |
| **Raw meat consumption** |  |  |  | 0.214 |
| No | 344 (96.1) | 339 (98.5) | 5 (1.5) |  |
| Yes | 14 (3.9) | 13 (92.9) | 1 (7.1) |  |
| **Processed/cured meat†** |  |  |  | 0.697 |
| Yes | 148 (41.8) | 145 (98.0) | 3 (2.0) |  |
| No | 206 (58.2) | 203 (98.5) | 3 (1.5) |  |
| **Raw vegetables †** |  |  |  | 0.443 |
| No | 39 (11.0) | 38 (97.4) | 1 (2.6) |  |
| Yes | 316 (89.0) | 312 (98.7) | 4 (1.3) |  |
| **Homegrown produce consumption †** |  |  |  | 0.680 |
| No | 271 (77.2) | 267 (98.5) | 4 (1.5) |  |
| Yes | 80 (22.8) | 79 (98.7) | 1 (1.3) |  |
| **Fruit consumption †** |  |  |  | 0.338 |
| No | 28 (7.9) | 27 (96.4) | 1 (3.6) |  |
| Yes | 328 (92.1) | 324 (98.8) | 4 (1.2) |  |
| **Milk consumption †** |  |  |  | 0.722 |
| No | 74 (20.8) | 73 (98.6) | 1 (1.4) |  |
| Yes | 282 (79.2) | 278 (98.6) | 4 (1.4) |  |
| **Raw milk consumption** |  |  |  | 0.903 |
| No | 352 (98.3) | 346 (98.3) | 6 (1.7) |  |
| Yes | 6 (1.7) | 6 (100) | 0 (0.0) |  |

*Media (SD). ** T-test. † Missing data. All analyses by Fisher's exact test.
